# Devastating Decline of Forest Elephants in Central Africa

**DOI:** 10.1371/journal.pone.0059469

**Published:** 2013-03-04

**Authors:** Fiona Maisels, Samantha Strindberg, Stephen Blake, George Wittemyer, John Hart, Elizabeth A. Williamson, Rostand Aba’a, Gaspard Abitsi, Ruffin D. Ambahe, Fidèl Amsini, Parfait C. Bakabana, Thurston Cleveland Hicks, Rosine E. Bayogo, Martha Bechem, Rene L. Beyers, Anicet N. Bezangoye, Patrick Boundja, Nicolas Bout, Marc Ella Akou, Lambert Bene Bene, Bernard Fosso, Elizabeth Greengrass, Falk Grossmann, Clement Ikamba-Nkulu, Omari Ilambu, Bila-Isia Inogwabini, Fortune Iyenguet, Franck Kiminou, Max Kokangoye, Deo Kujirakwinja, Stephanie Latour, Innocent Liengola, Quevain Mackaya, Jacob Madidi, Bola Madzoke, Calixte Makoumbou, Guy-Aimé Malanda, Richard Malonga, Olivier Mbani, Valentin A. Mbendzo, Edgar Ambassa, Albert Ekinde, Yves Mihindou, Bethan J. Morgan, Prosper Motsaba, Gabin Moukala, Anselme Mounguengui, Brice S. Mowawa, Christian Ndzai, Stuart Nixon, Pele Nkumu, Fabian Nzolani, Lilian Pintea, Andrew Plumptre, Hugo Rainey, Bruno Bokoto de Semboli, Adeline Serckx, Emma Stokes, Andrea Turkalo, Hilde Vanleeuwe, Ashley Vosper, Ymke Warren

**Affiliations:** 1 Global Conservation Program, Wildlife Conservation Society, Bronx, New York, United States of America; 2 School of Natural Sciences, University of Stirling, Stirling, Scotland, United Kingdom; 3 Department of Fish, Wildlife, and Conservation Biology, Colorado State University, Fort Collins, United States of America; 4 Save The Elephants, Karen, Nairobi, Kenya; 5 Lukuru Wildlife Research Foundation, Gombe, Kinshasa, Democratic Republic of Congo; 6 The Institute of Biodiversity and Ecosystem Dynamics, University of Amsterdam, Amsterdam, The Netherlands; 7 Ministère des Eaux, Forêts, Chasse et Pêche, Bangui, Central African Republic; 8 Beatty Biodiversity Centre, University of British Columbia, Vancouver, British Columbia, Canada; 9 Direction de la Gestion de la Faune et de la Chasse, Ministère des Eaux et Forêts, Libreville, Gabon; 10 Central Africa Regional Programme Office, World Wildlife Fund, Yaoundé, Cameroon; 11 The Jane Goodall Institute, Arlington, Virginia, United States of America; 12 Central Africa Program, Zoological Society of San Diego, Yaoundé, Cameroon; 13 Zoological Society of London, Regents Park, London, United Kingdom; 14 Behavioral Biology Unit, University of Liege, Liege, Belgium; 15 African Wildlife Foundation, Gombe, Kinshasa, Democratic Republic of Congo; Fordham University, United States of America

## Abstract

African forest elephants– taxonomically and functionally unique–are being poached at accelerating rates, but we lack range-wide information on the repercussions. Analysis of the largest survey dataset ever assembled for forest elephants (80 foot-surveys; covering 13,000 km; 91,600 person-days of fieldwork) revealed that population size declined by ca. 62% between 2002–2011, and the taxon lost 30% of its geographical range. The population is now less than 10% of its potential size, occupying less than 25% of its potential range. High human population density, hunting intensity, absence of law enforcement, poor governance, and proximity to expanding infrastructure are the strongest predictors of decline. To save the remaining African forest elephants, illegal poaching for ivory and encroachment into core elephant habitat must be stopped. In addition, the international demand for ivory, which fuels illegal trade, must be dramatically reduced.

## Introduction

The basic information required for effective conservation management of a species includes population status and distribution, identification and prioritization of threats, and trends in all of the above [1]. These metrics are the basis by which the IUCN Red List assesses the conservation status of species [2], and conservation policymakers and managers in the field decide on the management strategies which best serve the taxon in question. However, these requirements are notoriously difficult to ascertain and, therefore, lacking for numerous species including one of the world’s largest terrestrial mammals, the African forest elephant.

There are two distinct types of African elephants, often considered to be two species: savannah elephants *Loxodonta africana* (Blumenbach, 1797) and forest elephants *L. cyclotis* (Matschie, 1900). In 2003, the IUCN African Elephant Specialist Group (AfESG) listed them as subspecies *(L. a. cyclotis* and *L. a. africana,* respectively), due to perceived data gaps [3]. However, in 2008 they suggested that further research may reveal more than one African elephant species [4]. It was proposed that they should be considered two species on morphological grounds [5] and are considered as such by the Convention on Migratory Species [6]. Genetic evidence also supports this view [7]–[15]. The AfESG do, however, stress that it is important to recognize the different challenges to the conservation of forest and savannah elephants [3], [16].

African forest elephants have deep ecological differences from savannah elephants. They are highly frugivorous [17]–[20] and thus play an important role in one of Earth’s primary carbon-sequestering forests [17], [21], [22]. They can move great quantities of large seeds many kilometres from the parent tree [17] and are thus integral for maintaining forest structure and diversity. They also maintain [23], and possibly create, forest clearings in mineral-rich soil, on which a wide variety of African forest fauna are dependent [24], [25].

The history of African elephant abundance and distribution is strongly linked to the commerce in ivory, and their decline since the 1800s has been documented across the continent [26]–[28]. Even in the forests of Central Africa, a century ago, there were very few elephants remaining anywhere along the Gabonese coast, or around Brazzaville, in what is now the Republic of Congo [29]. It was thought that there was a slow decline in elephant populations during the 19^th^ century, flattening off in the first half of the 20^th^ century, and then a steep drop between 1950 and 1989 [28]. Modern African elephant density, based on data up to 2007, has recently been shown to be correlated with human factors rather than ecological factors [30].

The elephant subpopulation of Central Africa (which included some savannah populations in Chad and northern Cameroon) was recognized in 2008 as Endangered by the IUCN [4]. In 2010, the African Elephant Action Plan [31] drawn up by all of the African elephant range states, ranked poaching and illegal trade in elephant products as the top threat to elephants across the continent. In the last few years there have been very large and frequent ivory seizures in Africa and Asia, and the combination of seizure data analysed by the Elephant Trade Information System (ETIS) and of elephant carcass data documented and analysed by the Monitoring the Illegal Killing of Elephants (MIKE) programme demonstrate that the illegal trade is escalating [16], [32]–[36]. This increasing trade has been linked to increasing demand and value of ivory in China [37], [38]. The proportion of elephant carcasses found that had been killed illegally in 2010 was the highest on record [39] only to be exceeded by 2011 levels [16], [35]. Elephant meat is an important by-product, but ivory is the primary reason for elephant poaching [40]. It is now clear that elephants in general, and especially the elephants of Central Africa, are under serious threat [33] and that the poaching since 2011, may be at the level at which all elephant populations are in net decline [16], [31].

The scale of historical forest elephant decline, although substantial [28], [41], has been difficult to quantify due to a lack of comprehensive, range-wide information on distribution and density. Previous analyses, collected over a relatively short period and limited in geographic extent relative to their range, suggested a growing crisis for elephants in the Central African forests [42]. It is critical that a broader assessment is provided to understand range and demographic trends [16]. The Central African forest block covers about 95% of the current “known” and “possible” range of forest elephants [43]; the remaining 5% are in the forests of West Africa, to the west of the Cameroon-Nigeria border. We present the analysis of eighty surveys carried out over the nine-year period between 2002–2011 across the Central African forest block. The area stretches from the western Cameroon across to the eastern border of the Democratic Republic of Congo (DRC). The analysis responds to recent demands for a rigorous, range-wide assessment of forest elephant conservation status [16], [31]. Trends inferred from dung surveys are presented. In addition, landscape covariates correlated with dung density (a proxy for elephant density) were analyzed and results discussed with the aim of providing information to enhance effective conservation policy and management.

## Results

**Figure 1:**
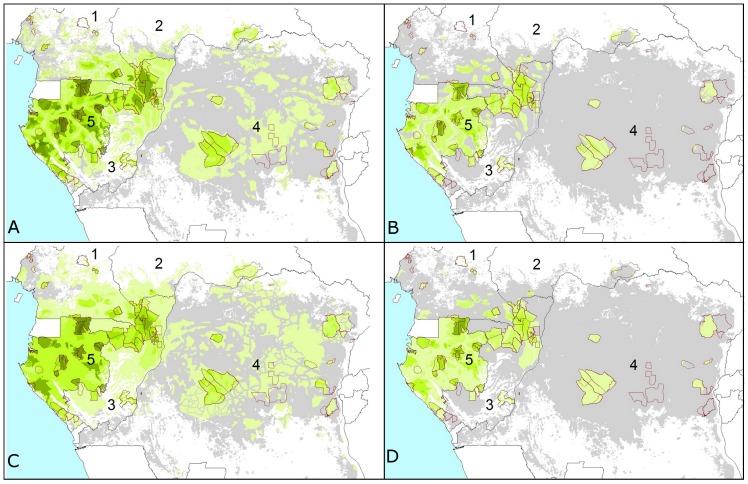
Elephant dung density and range reduction across the Central African forests. Predictions are shown for (A) 2002 and (B) 2011 for the model with variables: survey year∧, Human Influence Index***, corruption*** and the presence/absence of guards***, and (C) 2002 and (D) 2011 for the model with variables: survey year∧, proximity to road∧, human population density***, corruption*** and the presence/absence of guards*** (P-values are: ‘***’ <0.001 and ‘∧’ <0.1). Increasingly darker shades of green correspond to higher densities, grey represents extremely low elephant density range (the first interval: 0–100 elephant dung piles/km^2^) and white is non-habitat (80 survey sites outlined in red). Cutpoints are: 0; 100; 250; 500; 1,000; 1,500; 3,000; 5,000; and 7,500 dung piles/km^2^. Countries 1–5 are: Cameroon; Central African Republic; Republic of Congo; DRC; Gabon.

**Figure 2:**
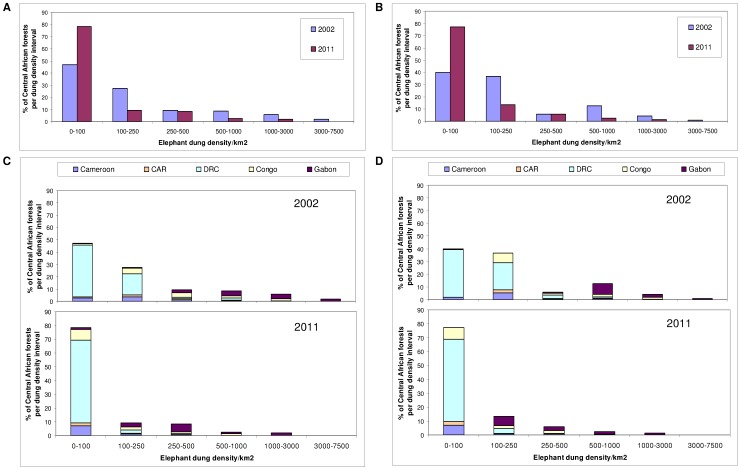
Estimated change in elephant dung density (/km^2^) distribution during 2002–2011 across the Central African forests. Results are shown as a percentage of the total area of potential elephant habitat overall (A & B) and by country (C & D) for the predictive model with variables: (A & C) survey year, Human Influence Index, corruption and the presence/absence of guards, and (B & D) survey year, proximity to road, human population density, corruption and the presence/absence of guards. The dung density (per km^2^) intervals are unequal and correspond to the following elephant population categories: extremely low density (0–100), very low (100–250), low (250–500), medium (500–1,000), high (1,000–3,000) and very high (3,000–7,500). With the loss of very high elephant populations in 2011, there is a significant shift into the lower density intervals over the nine years.

Our results demonstrate a widespread and catastrophic decline in numbers of forest elephants, in the order of 62%, and a corresponding range contraction of approximately 30%, during the nine-year period 2002–2011 represented by this study (Figs. 1 and 2; Tables S2 and S3). Forest elephants now have likely declined to extremely low density over 75% of their potential range (Tables S3, S6), and probably have been extirpated from large sections of this range. Considering 2002–2011 range contraction relative to elephant habitat per country, ca. 95% of DRC’s forests are likely to be almost empty of elephants, a country historically thought to have held the highest numbers (Table S3). About half of the surviving elephants are in Gabon, and under a fifth in DRC, despite these countries covering 13% and 62% of the total forest area, respectively (Table S6). In 2011, less than 2% of the Central African forest contained elephants at high density (Table S3). Even for Gabon, in 2011 high density populations were found in only 14% of the forest (a decline of over 18% between 2002 and 2011). No high density areas remained in DRC even in 2002.

### Correlates of Decline in Multi-variable Models

**Figure 3:**
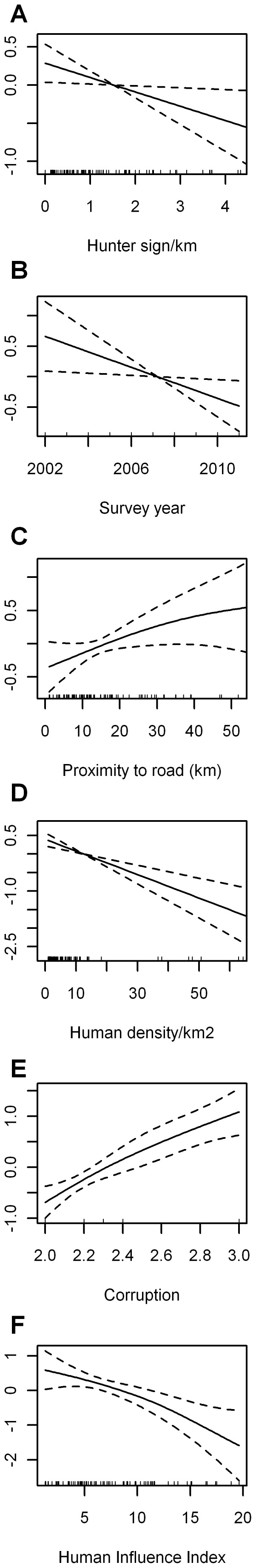
Estimated conditional dependence of elephant dung density for top-ranked multi-variable models including hunter sign. Results are shown for the top-ranked model with variables: (A) hunter sign*, (B) survey year*, (C) proximity to roads∧, (D) human population density***, (E) corruption*** (higher values = less corrupt) and presence/absence of guards***. Also shown is (F) the Human Influence Index (HII) for the model with proximity to road and human population density variables replaced by the HII, i.e. one of the top-ranking models with variables: hunter sign**, survey year*, HII*, corruption***, and presence/absence of guards***. P-value significance codes are: ‘***’<0.001, ‘**’<0.01, ‘*’<0.05, and ‘∧’<0.1. Plot components are: Estimates on the scale of the linear predictor (solid lines) with the y-axis scale for each variable selected to optimally display the results, confidence intervals (dashed lines), and explanatory variable values of observations with a focus on the core 95% of values for hunter sign, proximity to road and human population density (rug plot - short vertical bars along each x-axis showing the x value for each site).

**Figure 4:**
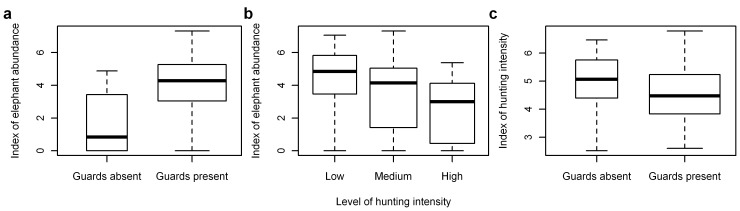
Boxplots of indices of elephant abundance and hunting intensity. Summaries shown are the natural logarithm of: (A) elephant dung encounter rate per 100 km grouped by the presence/absence of wildlife guards, (B) elephant dung encounter rate per 100 km grouped by the level of hunting intensity (group cutpoints are 0.6 and 1.75 hunter sign/km), and (C) hunter-sign frequency per 100 km grouped by the presence/absence of wildlife guards. Box-widths are proportional to the number of observations in each group.

**Figure 5:**
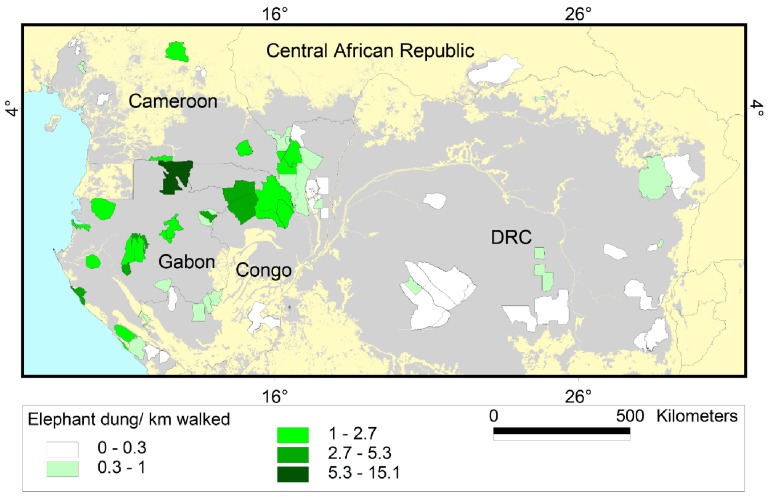
Encounter rate of elephant dung per kilometre. Results are shown for the 80 survey sites in Central Africa included in this study. Grey shading represents forest cover.

**Figure 6:**
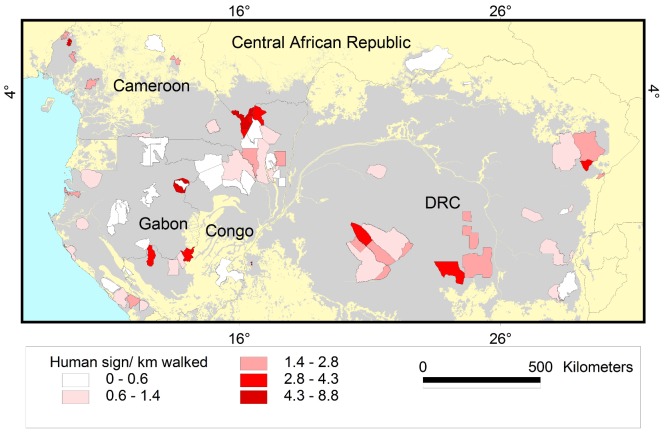
Encounter rate of hunter sign per kilometre. Results are shown for the 80 survey sites in Central Africa included in this study. Grey shading represents forest cover.

The overall top-ranked multi-variable model of elephant dung pile density by increasing Un-Biased Risk Estimator (UBRE) score included the explanatory variables: hunter-sign frequency, survey year, proximity to roads, human population density, corruption, and presence or absence of wildlife guards (Table S5, and Fig. 3). Site-specific dung-encounter rates and hunter-sign frequency were significantly negatively correlated–elephants occur where people do not–and both were strongly influenced by guard presence/absence (Figs. 4, 5, and 6). Survey year and corruption were included in almost all of the top-ranking models that included hunter sign. Models that included hunter-sign frequency were always better when considering UBRE score than otherwise identical models that excluded this variable and able to explain on average 50% of the variability in the data with satisfactory model fit diagnostics (Table S5, and Fig. 3). The top-ranking models without the hunter sign covariate were similar to each other in terms of UBRE score and were able to explain on average 45% of the variability in the data with satisfactory model fit diagnostics with models including the HII (Human Influence Index: [44]), in place of road proximity and human population density, generally a few percentage points lower; (Table S2, and Fig. S1). Again, survey year and corruption were included in almost all of these models. While hunter sign was clearly an important variable, it was one of the few for which data were collected directly during the surveys at each site (rather than extracting the information from GIS data layers, for example). Because it was site collected and not part of a global dataset such as the HII, it was not available at all locations across the Central African forests. Therefore, models containing hunter sign could not be used to produce predicted dung density surfaces and to estimate elephant range and abundance across the entire area of interest.

For the set of top-ranked models that used the variables across Central Africa, dung density was significantly higher at sites with wildlife guards and with a designated official protection status (Fig. 4). Dung density was inversely correlated with corruption as measured by Transparency International’s Corruption Perception Index (CPI) [45]; with more widespread distributions and higher densities in less corrupt countries: Gabon was significantly higher and DRC significantly lower than the roughly similar Cameroon, Central African Republic (CAR) and Republic of Congo (Congo) (Table S3). The regional proxy variables latitude and longitude appeared frequently among the top-ranking models and also captured significant variation. Longitude was the better covariate. In most of the models including either of these variables, these proxies indicated higher dung densities closer to the equator and significant decreases further east, which potentially represents site differences not accounted for by other variables, such as political instability in the Southeast (Eastern DRC) of the study area [46].

The inclusion of variables such as human population density, HII, and the presence/absence of wildlife guards always improved the UBRE score and were always statistically significant. Inclusion of variables such as proximity to roads, survey year and corruption in the models also improved the UBRE score, but these variables were occasionally non-significant. Either the HII or the combination of proximity to roads and human population density was used (with only human population density in some models), as the composite variable HII was highly correlated with the other two variables that comprise two of several variables used to generate the HII [44]. When considering the relative performance of the significantly correlated variables official protection and the presence/absence of guards (where official protection was low, there were no guards), the latter was much better in terms of improvements to the UBRE score and its effect on deviance explained, and was thus the preferred variable in top-ranking models. Corruption, as measured by the CPI was very highly correlated and almost identical to the country factor in terms of improvements to the UBRE score and its effect on deviance explained; with the added benefit of providing insights on how corruption, conceptually associated with poaching, may be influencing elephant distribution and density by country.

### Correlates of Decline in Single-variable Models

The single variable modelling results were similar to the multi-variable models, where all variables considered were significantly related to elephant dung density (Fig. S2 and Table S4). Univariate models with the variables longitude, country, corruption, and survey year were highly ranked, whereas the model with official protection received the lowest ranking (the UBRE scores for the remaining variables are also shown in Table S4). Univariate models predicted that dung density decreased by (i) 89% as hunter-sign frequency increased from zero to four per km, (ii) 85% when guards were absent, (iii) 30% or 76% as proximity to road decreased from 50 to 25 or zero km, (iv) 48%, 75% or 92% as human density increased from one to five, 10 or 20 people/km^2^, and (v) 17% for each unit increase in the HII. Among the survey site specific variables, human population density had the highest value for deviance explained, followed by the hunter-sign frequency, the presence/absence of guards, and the HII. For the remaining site specific variables (official protection status, proximity to roads, and survey year) the values were considerably smaller. The highest deviance explained corresponded to country-level variables, such as country itself and corruption. The proxy variable longitude also had one of the largest values for deviance explained, whereas latitude did not (Table S4).

### Predictive Modelling of Decline

We used the top-ranking multi-variable models with available regional data to predict forest elephant dung density across Central Africa (Fig. 1 and Table S2). We chose to highlight two models including predictor variables that elephants might be responding to directly, rather than latitude and longitude, so as to avoid using spatial location as a proxy for other processes. These models also include survey year as a covariate, which allows for predictions by year and comparisons over time. Results were consistent across models, and predicted dung density across Central Africa reflected the map of actual dung encounter rate (Fig. 5) and also most of the “Known” range described by the African Elephant Database (AED) [47]. Broadly speaking, whether using the HII or a combination of road proximity and human population density, the forested regions of Gabon, northern Congo, southwestern CAR and southeastern Cameroon contained the region’s highest elephant densities and almost all the nationally important elephant populations, while most of DRC, eastern Congo and southern CAR had very low densities (Fig. 1). The most country-specific important sites for elephants are as follows: in Gabon, most of the National Parks and their surroundings (often Forest Stewardship Council (FSC)-certified logging concessions), especially all of those in the centre and northeast of the country, plus a long section along the coast. In Congo, about half of the north of the country can be classed as an important elephant site, including not only the National Parks of Odzala and Nouabale-Ndoki (and the soon-to-be declared Ntokou-Pikounda National Park) but several huge areas of FSC certified timber concessions that connect and surround these Parks. In the Central African Republic, the Dzanga-Sangha National Park, and in Cameroon the whole of the southeast corner of the country, which includes three National Parks and large areas of FSC-certified logging concessions. Finally, the two significant sites in DRC are the Okapi Faunal Reserve and the Salonga National Park, with smaller but still significant numbers in some of the other forest areas (including one which may soon be gazetted as a protected area, known as the Tshuapa-Lomami area).

**Figure 7:**
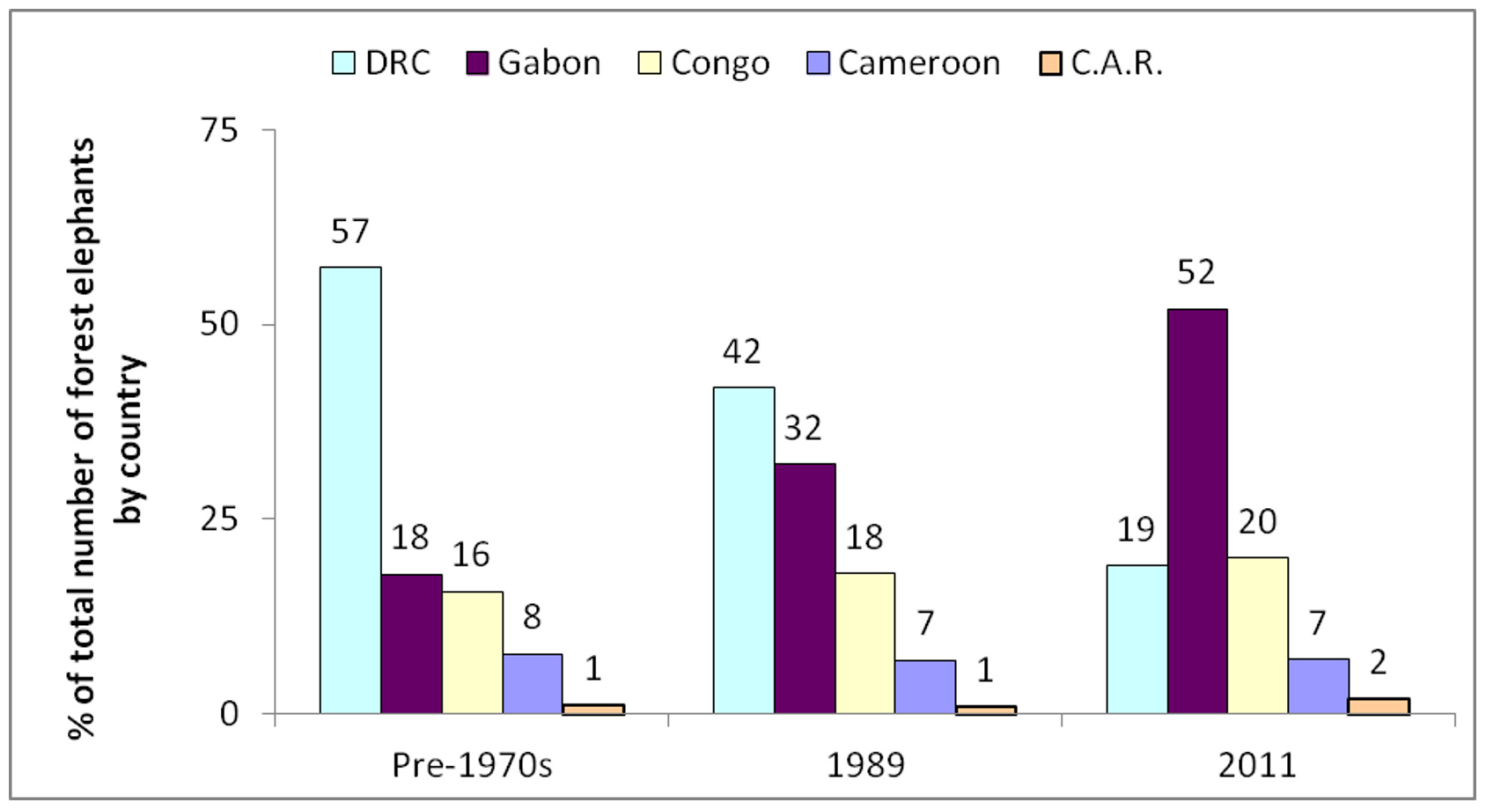
Percentage breakdown of the total number of forest elephants by country. Results are shown for 3 time periods: pre-1970s and 1989 [41] and 2011 (this study).

In areas where there is little or no poaching, elephant density is usually 0.5–1.0/km^2^ (data included in this study). Using a conservative density of 0.5 elephants/km^2^, historically the 2.2 million km^2^ Central African forest could have harboured over a million individuals [28], [41] (Fig. 7). Even in 1993, it was estimated that roughly half of this projected original population remained [41] (based on their model predictions). Our analysis suggests that in 2011 just 10% (ca. 100,000 individuals) still survive (99,869 with 95% bootstrapped confidence interval (49,867–187,340) for the predictive model shown in Fig. 1B). Gabon maintains 30–50% of its probable historical numbers; DRC only 1% - it was thought that DRC originally contained almost 60% of all forest elephants, and had 40% in 1989 [41].

## Discussion

Elephants have been recently extirpated from extensive areas of Africa [30], [34], [46], [47], [48] and even sites thought to be well-protected are no longer safe from ivory poaching [32]. Bouché et al.’s (2011) study examined the West and Central African savannahs, and showed that the once large savannah elephant populations had been reduced to several small pockets of a few hundred animals in many cases, with only about 7,000 individuals remaining in total. Shortly after that publication, in early 2012, several hundred elephants were killed in a matter of a few months, in the Park holding most of Cameroon’s savannah elephants [49], [50]; the poachers were well-armed and on horseback. In mid-November 2012, the same poachers were heading back to the same Park – but the Cameroon army were alerted before they arrived [51]. In February 2013, the Gabonese Government announced the loss of at least half of the elephants in Minkebe National Park; as many as 11,000 individuals may have been killed between 2004 and 2012 [52]. The rapid increases in demand for, and price of, ivory in China, and the ease of sale of ivory in China [37], [38], the persistent lack of effective governance in Central Africa [53] and a proliferation of unprotected roads that provide access to hunters [54], [55] combine to facilitate illegal ivory poaching, transport and trade. Forest elephant population and range will continue to decline unless conditions change dramatically.

Other threats and management issues also affect forest elephants. Unlike other tropical forests, deforestation is very low in Central Africa, although increasing [56], [57]. Nevertheless, land use pressure, habitat loss, and human-elephant conflict also threaten this species [16] and will likely increase as industrial agriculture, such as oil palm for biofuel production, develops in the near future in Africa in general and Central Africa in particular [58], [59]. While these management issues will likely increase with accelerating land use changes, the immediate, and very serious threat to the persistence of this species remains ivory poaching.

Our analysis identified several factors likely to contribute to decline and demonstrated the importance of law enforcement for persistence of elephants. Similar factors were also found to be important in recent analyses of a very different dataset- carcass data from the MIKE sites [16], [33] – where higher levels of elephant poaching, as expressed by the proportion of illegally killed elephants (PIKE) were associated with sites where law enforcement capacity was lower, and in countries with poor governance. Governance in our study was represented by the CPI [45], whereas the MIKE analysis up to 2009 [33] incorporated both CPI and several government effectiveness indicators used by the World Bank (which can be found in their website http://info.worldbank.org/governance/wgi/). However, in 2012, the MIKE analysis used only the CPI as the proxy for governance [16]. Because the CPI is strongly associated with other factors within countries (rule of law, governance, development), it may be considered as a proxy for overall functioning of civil society of each country, and indeed development variables associated with poverty were also found to be associated with PIKE in both 2009 and 2012 [16], [33]. A previous analysis using data from the African Elephant Database [60] suggesting a link between elephant decline and poor governance was criticized [61], because latitude was a better explanatory variable and the data were collected using different methods of varying quality [4], [47]. The more recent analysis using the 2007 AED showed that the “country” variable, a complex interaction of human development and governance factors, explained elephant density very well [30]. In contrast to the AED’s quite variable data, we used highly comparable data obtained within a single vegetation type (closed canopy forest). Corruption in general is increasingly a focus of international attention, whether in the wildlife realm [62]–[64] or more broadly [65].

Currently the Red List classifies African elephants (*L. africana*) as Vulnerable, and the Central African population as Endangered [4]. Current losses (62% between 2002–2011) combined with previous losses [28], [41] indicate a decline of more than 80% in less than two elephant generations, ca. 25 years [47]. The criterion for listing a species as Critically Endangered is when that species has declined by >80% in ten years, or three generations, whichever is the longer. If, conservatively, there were half a million forest elephants in the Congo Basin in 1937 (three elephant generations ago) then about 80% have now been lost. The causes of the decline are unlikely to abate in the short term, and indeed may worsen. This strongly suggests consideration of an uplisting of the Central African forest elephant subpopulation status to Critically Endangered, under the IUCN red list criteria A4b,d (population reduction, and current and projected levels of exploitation) [66].

Remaining large landscapes of major importance for elephants comprise national parks embedded in land-use matrices including logging concessions, where wildlife guards operate in both park and concession [67], [68]. However, current site-based interventions in the region are generally inadequate to protect elephants, because conservation budgets are below that needed to achieve management success [42], [69] and local interventions do not mitigate macro-scale threats (i.e. infrastructure development, governance issues, and ivory demand). Effective multi-level action is imperative to save forest elephants. We strongly agree with the recommendations of the African Elephant Action plan, of which the highest priority objective was the reduction of poaching and trade in elephant products.

In 2012, China submitted a document to CITES on how it will improve its internal ivory trade [70], as internal and international awareness of the problem grows [16], [35], [36], [71]. China’s wildlife officials, among others, attended a wildlife anti-trafficking workshop in Gabon in early 2012 [72]. At the 2012 World Conservation Congress, two specific resolutions were passed [73], [74] to enhance the protection of elephants both in the range states and in the ivory-consuming countries, and a specific wildlife-crime related resolution was passed at the same time [75]. In November 2012, the US State department clearly outlined a zero-tolerance approach to wildlife crime [76], [77] and many governments, INTERPOL, the World Customs Association and others are collaborating in international efforts to curb ivory (and other wildlife product) trafficking [63], [78]- partly for the wildlife itself, and also because the strong links with global organised crime and security are recognised [64], [79]. These diplomatic efforts are critical, but we emphasize the importance of *in situ* enforcement investment to protect the remaining populations of this species. However, curbing demand for ivory is key, if forest elephants are to survive.

## Materials and Methods

### Ethics Statement

All research was conducted using observation of indirect signs of forest elephants (dung).

### Data Collection and Standardization

We modelled temporal and spatial trends using data collected during 13,000 km of elephant-dung foot-surveys in 80 sites during 91,600 person-days from 2002 to 2011 (Fig. S3 shows temporal coverage). Field protocol followed the standardized 2003 methods of the Monitoring the Illegal Killing of Elephants (MIKE) [80] program of the Convention on International Trade in Endangered Species (CITES). Surveys covered the five countries holding the majority (95%) [43] of extant forest elephant range: Cameroon, Central African Republic (CAR), Republic of Congo (Congo), Democratic Republic of Congo (DRC) and Gabon, across 257,145 km^2^ (about 12% of Central Africa’s forests; Table S1, Figs. 5 and 6). There were 9, 5, 25, 22 and 19 surveys in each country with ca. 11%, 2%, 26%, 43% and 18% of total effort (13,000 km) and ca. 4%, 6%, 32%, 41% and 17% of the total area covered (257,145 km^2^). For sites surveyed more than once, only the most recent data was used. In just over 25% of sites surveyed wildlife guards were absent.

All surveys were carried out independently for site-based or landscape-based conservation needs. Limited resources for these purposes resulted in surveys being restricted to areas known or suspected to harbour wildlife, but with very variable elephant densities. Over half of the surveys were of existing or prospective protected areas, and the rest were areas in logging concessions, or with potential for wildlife conservation. Although there were some sites where elephant populations were known to be very low, few sites thought to be completely devoid of them were surveyed. Survey data was obtained across the range of values for each of the covariates considered in the analysis.

Either standard systematic line-transect distance sampling surveys (perpendicular distance to each dung pile recorded) [81], or systematic reconnaissance surveys [82] (elephant dung only recorded within a metre of the centre line) were walked. Both transect and recce survey designs allowed for random placement of the sampling units being drawn up using Distance software [83], and orientation of both transects and recces were perpendicular to roads, and major rivers to potentially improve precision. Usually at least 15 transects per stratum were used; usually more, giving reasonable replication to ensure a representative sample was obtained. Transects and recces were usually placed systematically with a random start across the entire area surveyed. At some sites both recces and transects were walked; we have used only the transect data for these sites. Data from recce surveys were used when straight lines were walked, thereby ensuring minimal bias. Other data from less strict recces (where roads or elephant paths might have been used) were not used in the analysis. Most recces were from areas known to have high hunting pressure (and thus low wildlife density). This is because transects are much more expensive to implement than recces. Occasionally, recces were done in areas where resources for a transect survey were not available at the time. Transect data were truncated to one metre of the centre line using the Distance software and the resulting plot checked to ensure that detection was 100% within that distance [83]. For reconnaissance data, detectability of dung piles was assumed certain within the narrow sampling strip (one metre each side of the observer).

### Data Analysis

#### Statistical modeling

We assessed known or suspected drivers of elephant density and distribution [16], [17], [30], [33], [54], [55] using Generalized Additive Models (GAMs) [84] due to their flexibility and capacity for non-linear responses.

The standardized response variable was elephant dung pile counts within one metre of the centre line, adjusted for survey effort. GAMs were fit to elephant dung pile count data of the form:
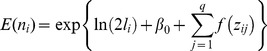
where for the *i* ^th^ survey site:_
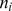
_ denotes number of elephant dung piles detected, 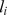 aggregate survey effort, 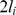 area effectively surveyed, 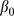 the intercept, and 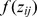 a smooth function of the *j* ^th^ explanatory variable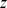. By including area surveyed as an offset term in the model, elephant dung density is in effect being modelled. A negative binomial distribution was used to deal appropriately with severe over-dispersion in the count data. The scale parameter theta of the negative binomial was treated as unknown and an interval of (1,3) over which to search for theta was specified. Thin plate regression splines were used to fit the smooth functions, where the ‘performance iteration’ method was used for smoothing parameter estimation. To avoid overfitting, given the limited number of data points (80 survey sites), the gamma parameter was set to a value of 1.4 for all models, which forced the model to be smoother than it might otherwise have been [84]. With the limited number of data points (80 sites across years) it was not possible to account for the nested nature of sites within countries by means of a hierarchical model structure; instead country was simply included as a factor variable. The models were fit in R [85] using the *mgcv* package.

Competing models, i.e. those with different covariates, were ranked by increasing Un-Biased Risk Estimator (UBRE) criterion [84]. The significance of explanatory variables (based on the P-values returned for each of the terms in the GAM), percent deviance explained by the model and model fit diagnostics (Normal Q-Q, residuals vs. linear predictor, histogram of residuals, response vs. fitted values) were also considered [84]. Model selection was based on the UBRE criterion [84].

Models where survey year was modelled by country served to examine country-specific changes over the period 2002–2011. There were indications of country specific changes over time (Fig. S4 shows how the decline in DRC is potentially more extreme than in Gabon, for example). However, given the sparseness of the time series for Cameroon and in particular CAR and the lack of data points for Congo and DRC at the beginning of the period (Fig. S3), we did not incorporate country specific changes over time in the final models used to predict dung density across the Central African forests. Instead we restricted our predictions to models with the same smooth function for temporal change across the Central African forests.

Given the similarity in UBRE scores for the top models, we estimated elephant dung density using each of them (Table S2). The bootstrapped 95% confidence intervals are also shown. The confidence limits are wide and the percent coefficients of variation were frequently larger than 100 (this was particularly the case for models including HII with the exception of models HII 3 and HII 5 in 2011). When models contained survey year (the proxy for temporal change), we estimated elephant dung density in both 2002 and in 2011, rather than just obtaining an average for the 2002–2011 period, which allowed us to calculate rates of decline and percent range loss from these models; estimates of the percentage of extremely low elephant density range overall and by country for 2002 and 2011 are also given (Table S3).

Variance and percentile confidence intervals of elephant abundance estimates were estimated using a combination of nonparametric and parametric bootstrapping [86]. A total of 999 bootstraps were conducted during which replicate survey sites, assumed to be independently and identically distributed, were resampled at random and with replacement until each bootstrap resample was the same size as the original number of 80 survey sites (nonparametric component). Dung abundance estimates were obtained from these resampled data conditioned on the original model fit. Dung abundance estimates were converted to elephant abundance by applying conversion factors (described below) with associated total variance obtained by incorporating the variance associated with the conversion factors. During each iteration of the bootstrap routine, conversion factor values were generated from a normal distribution with mean equal to the estimated value of the conversion factor and the variance equal to the squared value of the associated standard error (parametric component). Estimates of elephant numbers were ordered from smallest to largest and the 25^th^ and 975^th^ value was used to define the percentile confidence interval. The coefficient of variation was obtained by dividing the square root of the variance of the abundance estimates from the resampled data predictions by the mean of those abundance estimates.

We defined extremely low density areas where dung density fell between 0–100 dung piles/km^2^ (in practice this approximates to >0.1 dung pile encountered per km walked) based on knowledge of areas within Central Africa which have extremely few or no elephants remaining (in part relying on design-based estimates of dung abundance). All of the areas where we already knew that there were extremely few or no elephants (from historical surveys, from some of these surveys included here, or from other surveys not included in this dataset), such as the majority of the southern Republic of Congo, and the majority of the forests in western Cameroon, fell into this density class, giving us confidence in the model’s ability to predict elephant range where there are almost no elephants left.

#### Conversion factors

Dung density estimates were converted to elephant numbers using estimated production and decay rates since actual rates are notoriously difficult to collect ([87]) and were not available at every site. A production rate of 19.77 dung piles/day (standard error (SE) = 0.23) [88], also suggested by the rainfall regime of much of the area [89], was used. The same rate was also used to assess historical forest elephant loss up to 1989 [41], ensuring comparative differences were not a function of this model assumption. To ensure that the decay rate used in the conversion was representative of our sites that ranged widely in space and time, we used the mean (81.82 days, SE = 6.68 days) of fourteen estimates of dung disappearance time for different seasons, habitats and sites. No particular geographical pattern for decay rate from west to east across the basin was evident in these studies, and the associated variance was low enough to make us more confident in our application of this as a standard conversion factor, whilst recognising that there is variation within decay rates associated with season, sunspots, and rainfall [90], [91], [92]. To convert dung to elephant density, only dung piles not in a late stage of decomposition (“class E” of [93] are generally included. For this dataset dung density was reduced by 32.1% (SE = 3.7%), the mean percent of dung piles classified as “E”, before estimating elephant density.

#### Explanatory variables

Explanatory variables used in the GAM analysis were (Table S4) [94]: (i) *site-level* at the scale of individual sampling units, (ii) *country-level* (including country itself), and (iii) *regional proxies* latitude and longitude to capture possible geographical gradients in density not captured by other variables. Variables were either recorded at each survey site (hunter-sign frequency, survey year, presence/absence of guards), retrieved from reports (official protection reflecting the degree of potential protection) and online databases (Transparency International’s Corruption Perceptions Index [45], or from GIS data layers (distance-based for poacher access, i.e., proximity to major roads; pressure-based for poacher numbers, i.e., human population density [95], the Human Influence Index [44]. Square root transformations for hunter-sign frequency and human population density were considered due to possible undue leverage from the few high values. The predicted likely influence on elephant density for each of the explanatory variable is given (Table S4). Assumptions implicit in the choice of these variables were based on previous work [16], [17], [33], [54], [55]. We assumed that both direct hunting pressure (as measured by encounter rate of hunter sign) and measures of human population density and activity (as measured by distance to the nearest road, human population density, and the human influence index) results in elephants moving away from human-dominated areas and/or being killed by poachers. We assumed that official protection of a site (such as National Park status) would reflect real protection, in other words that elephants would be more likely to be at higher densities in such sites. We assumed that if guards were present at a site, that they were actually effective in deterring poaching. We assumed that our measure of governance (CPI) reflected the suite of social, economic, and development factors associated with each country; governance and development had previously been shown to be associated with elephant poaching by the two MIKE analyses in 2009 and 2012 [16], [33].

Pearson's product-moment correlation tests (two-sided) with null hypothesis that true correlation is equal to zero were conducted for each pair-wise combination of explanatory variables considered. Variables were considered significantly correlated at the 5% level. Correlations between variables were taken into account to avoid the inclusion of highly correlated variables in the same model. Model prediction was limited to Central African forested regions, including swamp forest [96], in the five countries with survey sites. GIS grids were created at a resolution of approximately 1x1 km^2^, and prediction was carried out at the same resolution.

#### Reporting results

Generally, averaged estimates from the set of top-ranking predictive models were given. Potential elephant range was defined by forest cover. Elephant range and high density elephant areas were estimated as the aggregate of areas with >100 and >1,000 elephant dung piles/km^2^, respectively.

## Supporting Information

Figure S1 **Estimated conditional dependence of elephant dung density for top-ranking multi-variable models without hunter-sign used for prediction across the Central African forests, using the variables available across Central Africa either as GIS layers or in country-specific databases.** Plots shown are for models with variables (A) survey year∧, Human Influence Index***, and corruption***, and (B) survey year∧, proximity to roads∧, human population density***, and corruption***. Presence/absence of wildlife guards was also included as a factor covariate in both models and dung density was significantly more - P<0.001 - at sites where guards were present. P-value significance codes are: ‘***’<0.001 and ‘∧’<0.1. Plot components are: Estimates on the scale of the linear predictor (solid lines) with the y-axis scale for each variable selected to optimally display the results, confidence intervals (dashed lines), and explanatory variable values of observations with a focus on the core 95% of the data for proximity to road and human population density (rug plot - short vertical bars along each x-axis).
(PDF)

Figure S2 **Estimated conditional dependence of elephant dung density for single variable models.** Results are shown for (A) hunter sign***, (B) survey year**, (C) proximity to roads*, (D) human population density***, (E) Human Influence Index***, (F) official protection*** (higher values = less protected), (presence/absence of wildlife guards is a factor covariate and thus not shown here, however, dung density was significantly higher - P<0.001 - at sites where guards were present), (G) corruption*** (higher values = less corrupt), (H) latitude*, and (I) longitude***. P-value significance codes are: ‘***’ <0.001, ‘**’ <0.01, and ‘*’ <0.05. Plot components are: Estimates on the scale of the linear predictor (solid lines) with the y-axis scale for each variable selected to optimally display the results, confidence intervals (dashed lines), explanatory variable values of observations with a focus on the core 95% of values for a, c and d (rug plot - short vertical bars along each x-axis).
(PDF)

Figure S3 **The number of survey sites per country by survey year.** Results are shown for the 80 survey sites in Central Africa.
(PDF)

Figure S4 **Estimated conditional dependence of elephant dung density considering survey year by country for a multi-variable models including hunter sign.** Survey year by country focusing on the Democratic Republic of Congo (DRC) and Gabon for the model with variables hunter sign*, survey year by country*, proximity to roads, human population density***, corruption*** and presence/absence of guards*** (dung density was significantly more - P<0.001 - at sites where guards were present). P-value significance codes are: ‘***’<0.001, ‘**’<0.01, ‘*’<0.05, and ‘∧’<0.1. Plot components are: Estimates on the scale of the linear predictor (solid lines) with the y-axis scale for each variable selected to optimally display the results, confidence intervals (dashed lines), and explanatory variable values of observations (rug plot - short vertical bars along each x-axis).
(PDF)

Table S1 **Details of the 80 survey sites included in the analysis.**
(PDF)

Table S2 **Analysis results for top-ranking predictive models (excluding hunter sign as an explanatory variable), which included (a) the Human Influence Index (HII), or (b) human population density and proximity to road (SPD).** Details of the variables included in each model are given and percent deviance explained and UBRE score value. Estimated average elephant dung density (/km^2^) from model predictions across the Central African forests and bootstrapped 95% confidence intervals are shown. If the model included the survey year variable then prediction is for the endpoints of the time series (2002 and 2011); otherwise the prediction can be interpreted as an average over the 2002–2011 time period. Also shown for the models that permit temporal prediction is the overall percent decline and overall percent range loss for the period 2002–2011 (elephants are assumed to be absent when dung density falls below 100 elephant dung piles/km^2^; see Table S3 and Figure 2 for details, including a breakdown by country).
(PDF)

Table S3 **Estimates of percentage extremely low density** **elephant range across the Central African forests and by country (relative to each country’s forested area) for 2002 and 2011 for the top-ranking predictive models, which included the survey year variable.** Elephants are assumed to be almost absent when dung density falls below a threshold value of 100 elephant dung piles/km^2^. Also shown are estimates of the percentage of potential habitat at high elephant density (defined as >1,000 elephant dung piles/km^2^). The average across all models for 2002 and for 2011 is shown, as well as the range Table S6 for a breakdown of forest cover by country.
(PDF)

Table S4 **Description of spatial variables, data source, method of calculation, likely influence on elephant density, UBRE score and deviance explained for the single variable models.**
(PDF)

Table S5 **Analysis results for top-ranking models which included the hunter sign variable.** Hunter sign was not included in the predictive model across the Central African forests, as it was unavailable at that scale.
(PDF)

Table S6 **Estimated forest cover by country as defined by Iremonger et al. (1997)** [**96**] **.**
(PDF)
