# Supplementary material for: Devastating Decline of Forest Elephants in Central Africa
Source: PLoS One. 2013 Mar 4;8(3):e59469. doi: 10.1371/journal.pone.0059469 (PMC3587600; doi:10.1371/journal.pone.0059469)
Supplement: Figure S1 — Estimated conditional dependence of elephant dung density for top-ranking multi-variable models without hunter-sign used for prediction across the Central African forests, using the variables available across Central Africa either as GIS layers or in country-specific databases. Plots shown are for models with variables (A) survey year∧, Human Influence Index***, and corruption***, and (B) survey year∧, proximity to roads∧, human population density***, and corruption***. Presence/absence of wildlife guards was also included as a factor covariate in both models and dung density was significantly more - P<0.001 - at sites where guards were present. P-value significance codes are: ‘***’<0.001 and ‘∧’<0.1. Plot components are: Estimates on the scale of the linear predictor (solid lines) with the y-axis scale for each variable selected to optimally display the results, confidence intervals (dashed lines), and explanatory variable values of observations with a focus on the core 95% of the data for proximity to road and human population density (rug plot - short vertical bars along each x-axis). (PDF) [file pone.0059469.s001.pdf]

Devastating Decline of Forest Elephants in Central Africa - Supplementary Figures

A

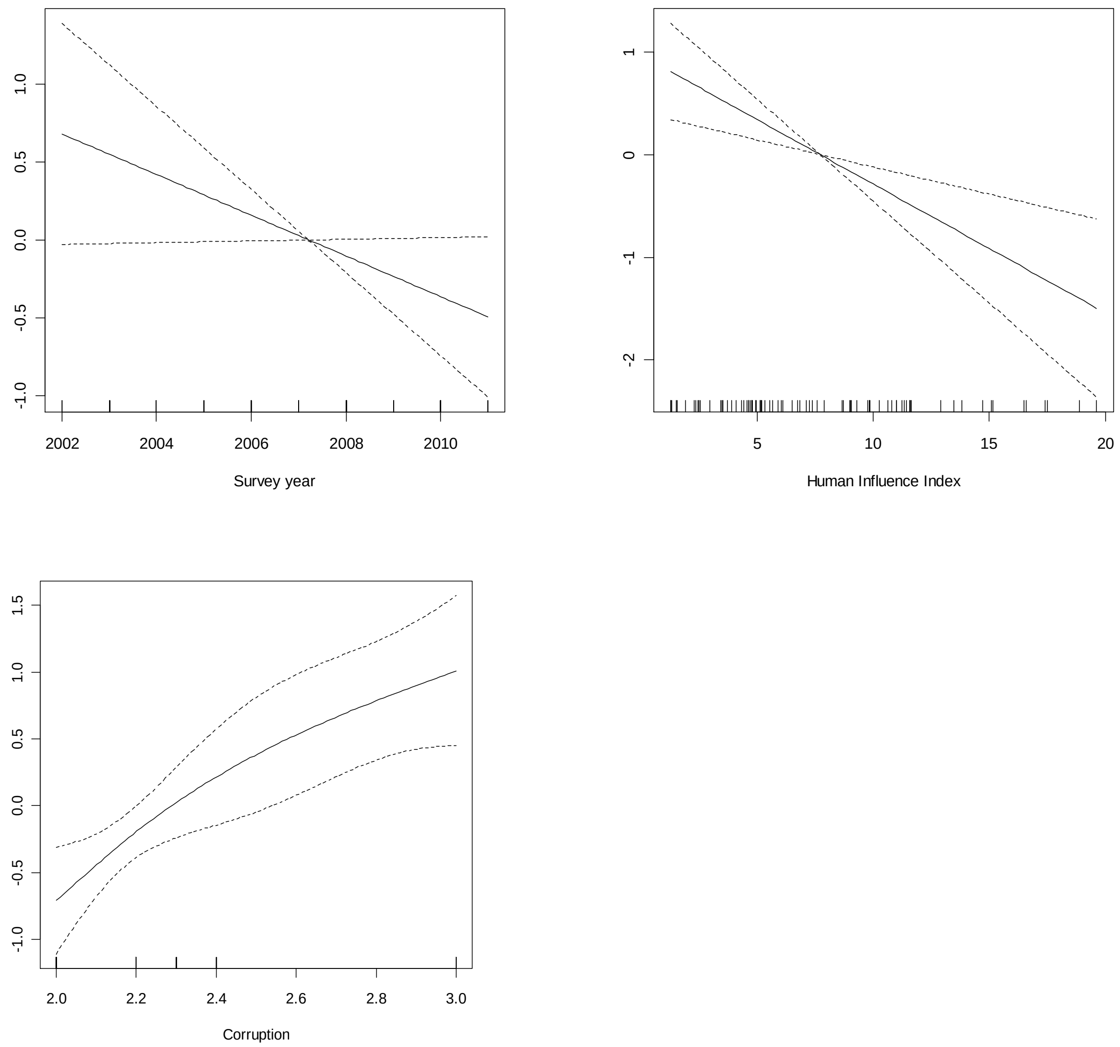

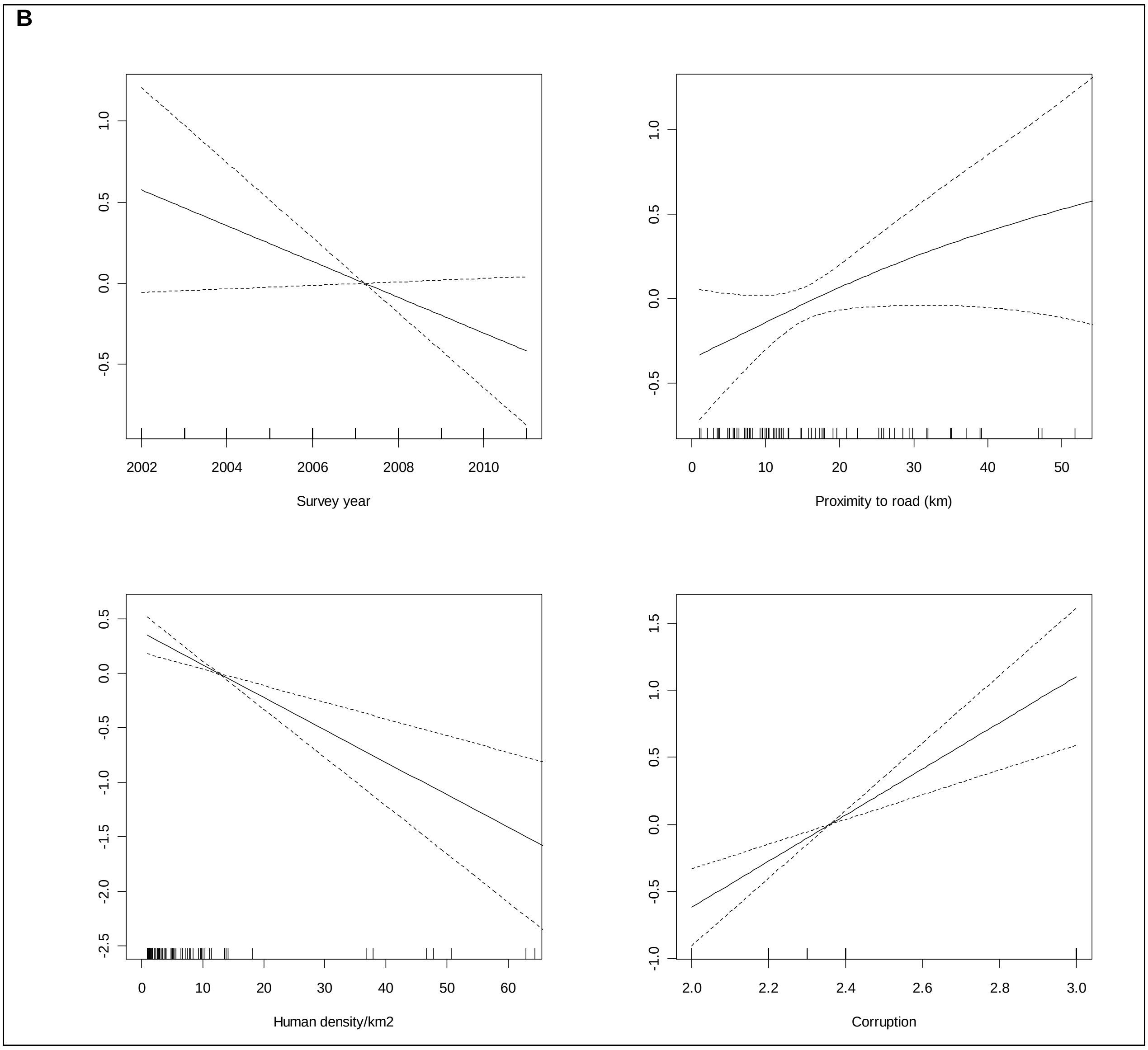

Fig. S1. Estimated conditional dependence of elephant dung density for top-ranking multi-variable without hunter-sign models used for prediction across the Central African forests, using the variables available across Central Africa either as GIS layers or in country-specific databases.

Plots shown are for models with variables (A) survey year^, Human Influence Index***, and corruption***, and (B) survey year^, proximity to roads^, human population density***, and corruption***. Presence/absence of wildlife guards was also included as a factor covariate in both models and dung density was significantly more - P < 0.001 - at sites where guards were present. P-value significance codes are: '***'<0.001 and '^'<0.1. Plot components are: Estimates on the scale of the linear predictor (solid lines) with the y-axis scale for each variable selected to optimally display the results, confidence intervals (dashed lines), and explanatory variable values of observations with a focus on the core 95% of the data for proximity to road and human population density (rug plot - short vertical bars along each x-axis).
