# Supplementary material for: Devastating Decline of Forest Elephants in Central Africa
Source: PLoS One. 2013 Mar 4;8(3):e59469. doi: 10.1371/journal.pone.0059469 (PMC3587600; doi:10.1371/journal.pone.0059469)
Supplement: Figure S2 — Estimated conditional dependence of elephant dung density for single variable models. Results are shown for (A) hunter sign***, (B) survey year**, (C) proximity to roads*, (D) human population density***, (E) Human Influence Index***, (F) official protection*** (higher values = less protected), (presence/absence of wildlife guards is a factor covariate and thus not shown here, however, dung density was significantly higher - P<0.001 - at sites where guards were present), (G) corruption*** (higher values = less corrupt), (H) latitude*, and (I) longitude***. P-value significance codes are: ‘***’ <0.001, ‘**’ <0.01, and ‘*’ <0.05. Plot components are: Estimates on the scale of the linear predictor (solid lines) with the y-axis scale for each variable selected to optimally display the results, confidence intervals (dashed lines), explanatory variable values of observations with a focus on the core 95% of values for a, c and d (rug plot - short vertical bars along each x-axis). (PDF) [file pone.0059469.s002.pdf]

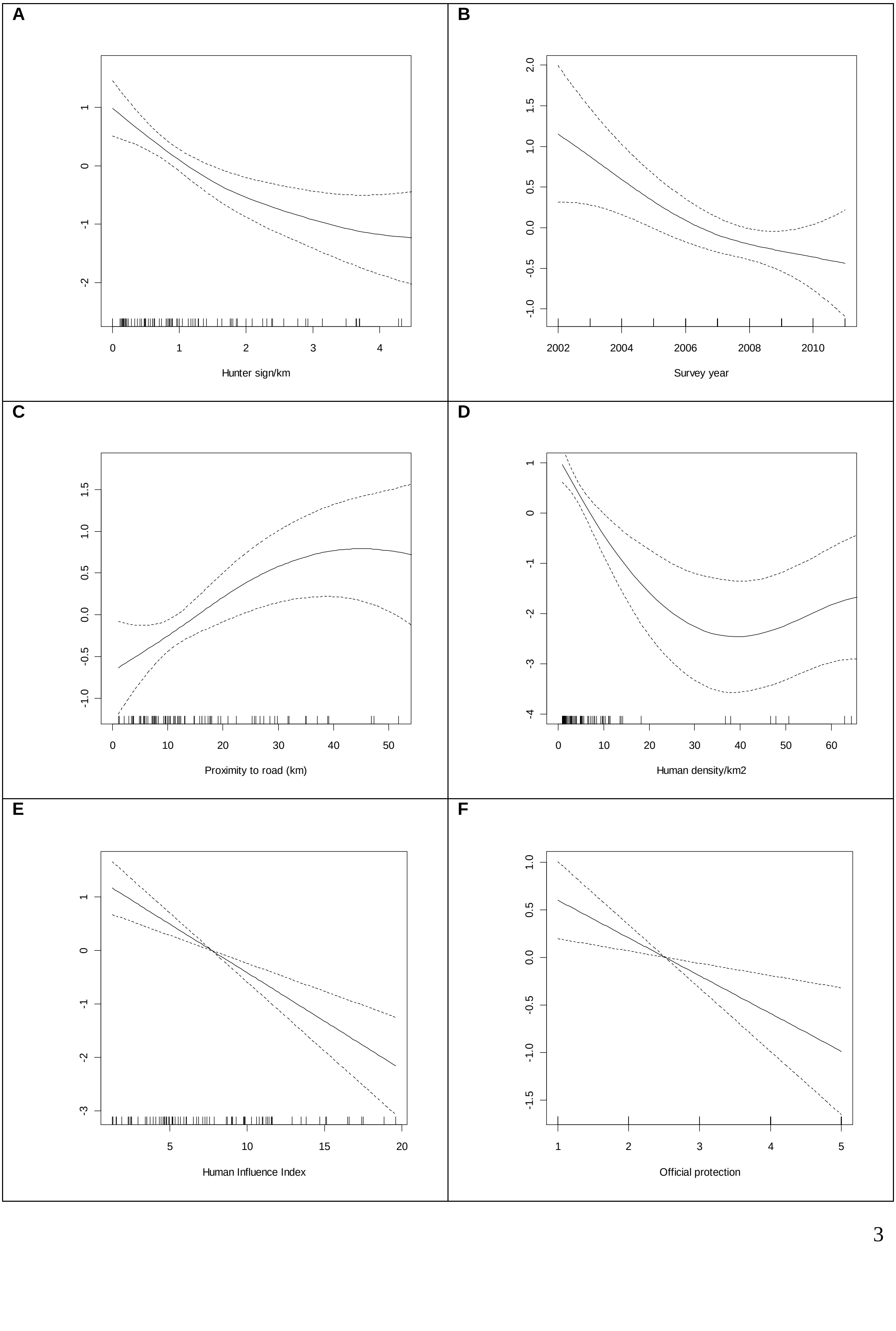
A
Hunter sign/km
B
Survey year
C
Proximity to road (km)
D
Human density/km2
E
Human Influence Index
F
Official protection

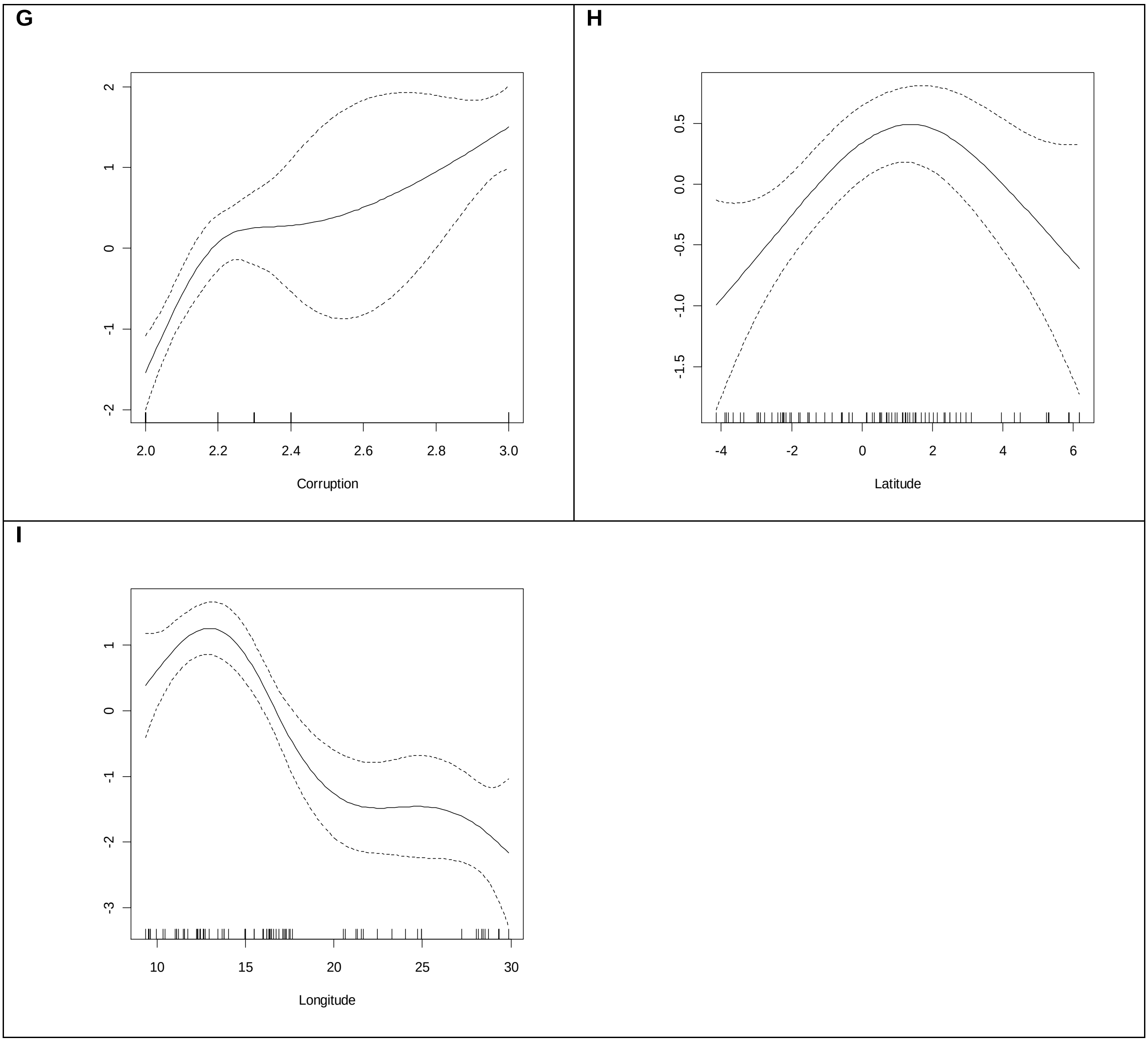

Fig. S2. Estimated conditional dependence of elephant dung density for single variable models.

Results are shown for (A) hunter sign***, (B) survey year**, (C) proximity to roads*, (D) human population density***, (E) Human Influence Index***, (F) official protection*** (higher values = less protected), (presence/absence of wildlife guards is a factor covariate and thus not shown here, however, dung density was significantly higher - $P < 0.001$ - at sites where guards were present), (G) corruption*** (higher values = less corrupt), (H) latitude*, and (I) longitude***. P-value significance codes are: '***' < 0.001, '**' < 0.01, and '*' < 0.05. Plot components are: Estimates on the scale of the linear predictor (solid lines) with the y-axis scale for each variable selected to optimally display the results,, confidence intervals (dashed lines), explanatory variable values of observations with a focus on the core 95% of values for a, c and d (rug plot - short vertical bars along each x-axis).
