# Supplementary material for: Devastating Decline of Forest Elephants in Central Africa
Source: PLoS One. 2013 Mar 4;8(3):e59469. doi: 10.1371/journal.pone.0059469 (PMC3587600; doi:10.1371/journal.pone.0059469)
Supplement: Figure S3 — The number of survey sites per country by survey year. Results are shown for the 80 survey sites in Central Africa. (PDF) [file pone.0059469.s003.pdf]

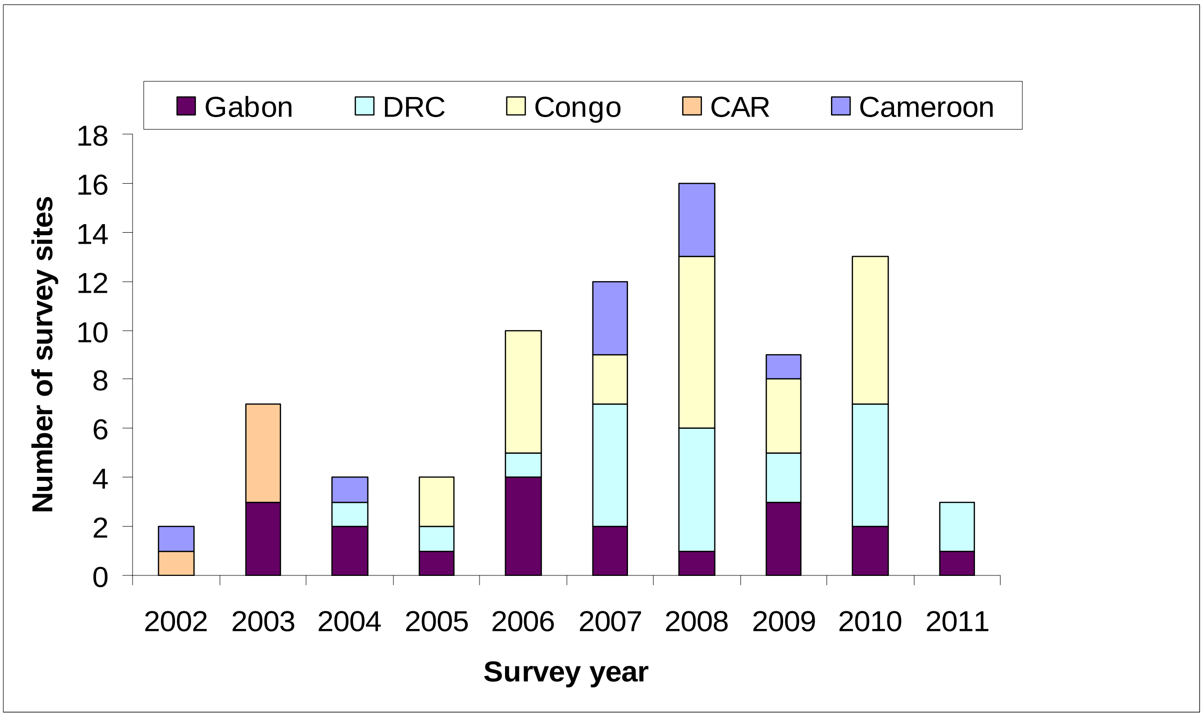

Fig. S3. The number of survey sites per country by survey year.

Results are shown for the 80 survey sites in Central Africa.
