# Supplementary material for: Devastating Decline of Forest Elephants in Central Africa
Source: PLoS One. 2013 Mar 4;8(3):e59469. doi: 10.1371/journal.pone.0059469 (PMC3587600; doi:10.1371/journal.pone.0059469)
Supplement: Figure S4 — Estimated conditional dependence of elephant dung density considering survey year by country for a multi-variable models including hunter sign. Survey year by country focusing on the Democratic Republic of Congo (DRC) and Gabon for the model with variables hunter sign*, survey year by country*, proximity to roads, human population density***, corruption*** and presence/absence of guards*** (dung density was significantly more - P<0.001 - at sites where guards were present). P-value significance codes are: ‘***’<0.001, ‘**’<0.01, ‘*’<0.05, and ‘∧’<0.1. Plot components are: Estimates on the scale of the linear predictor (solid lines) with the y-axis scale for each variable selected to optimally display the results, confidence intervals (dashed lines), and explanatory variable values of observations (rug plot - short vertical bars along each x-axis). (PDF) [file pone.0059469.s004.pdf]

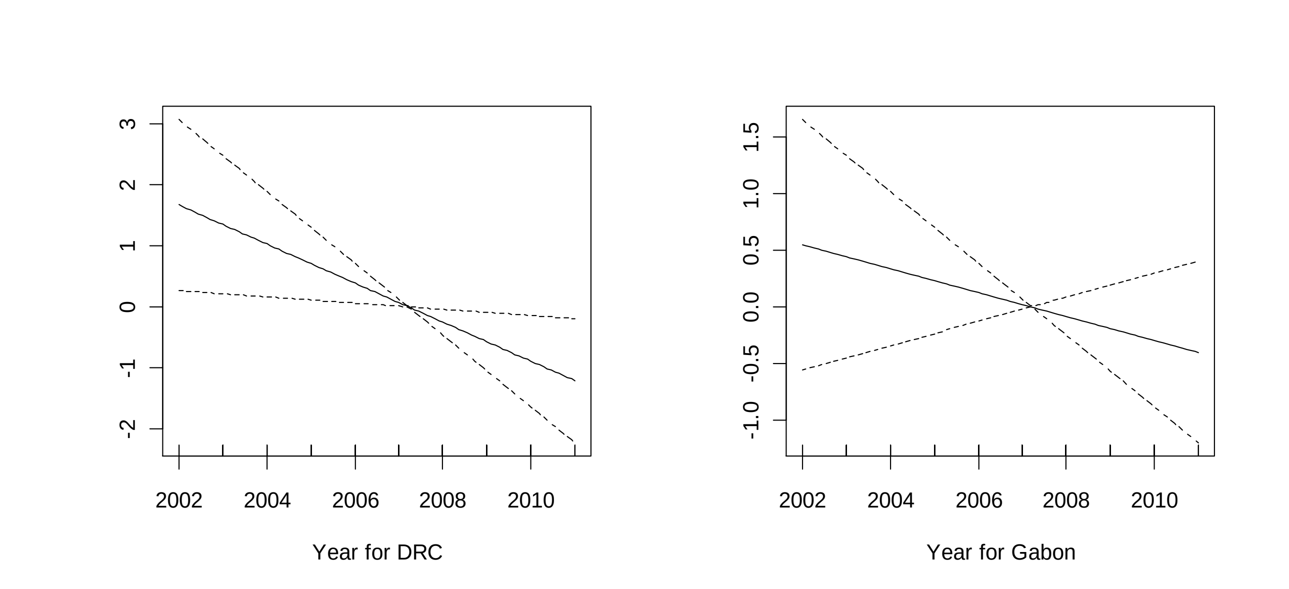

Fig. S4. Estimated conditional dependence of elephant dung density considering survey year by country for a multi-variable models including hunter sign.

Survey year by country focusing on the Democratic Republic of Congo (DRC) and Gabon for the model with variables hunter sign*, survey year by country*, proximity to roads, human population density***, corruption*** and presence/absence of guards*** (dung density was significantly more - P < 0.001 - at sites where guards were present). P-value significance codes are: '***'<0.001, '**'<0.01, '*'<0.05, and '^'<0.1. Plot components are: Estimates on the scale of the linear predictor (solid lines) with the y-axis scale for each variable selected to optimally display the results, confidence intervals (dashed lines), and explanatory variable values of observations (rug plot - short vertical bars along each x-axis).
