# Supplementary material for: Devastating Decline of Forest Elephants in Central Africa
Source: PLoS One. 2013 Mar 4;8(3):e59469. doi: 10.1371/journal.pone.0059469 (PMC3587600; doi:10.1371/journal.pone.0059469)
Supplement: Table S1 — Details of the 80 survey sites included in the analysis. (PDF) [file pone.0059469.s005.pdf]

Table S1. Details of the 80 survey sites included in the analysis.

Country	Survey Site	Area (km^2)	Survey Year	Elephant Dung Piles (within 1m)	Effort (km)	Hunter Sign (/km)	Method	Source	Guards present
Cameroon	Banyang Mbo	667	2007	22	43.70	2.00	Transects	1	Yes
Cameroon	Boumba Bek	2,383	2004	78	47.00	0.82	Transects	2/ 3	Yes
Cameroon	Deng Deng NP & UFA adjoining	778	2009	3	131.44	1.87	Transects	1	Yes
Cameroon	Ebo	1,417	2008	93	385.00	3.70	Recces	4	Yes
Cameroon	Mbam core	1,433	2008	303	114.00	0.63	Transects	1	Yes
Cameroon	Mbulu	200	2008	0	96.00	0.89	Recces	1	Yes
Cameroon	Mengame	1,151	2002	220	142.50	1.28	Transects	5	Yes
Cameroon	Mone	452	2007	1	188.00	6.42	Recces	1	
Cameroon	Takamanda NP	676	2007	17	232.00	1.85	Recces	1	Yes
CAR	Bangassou	10,000	2003	4	14.00	0.50	Transects	3/ 6	
CAR	Dzanga Sangha SR	3,101	2003	15	24.00	6.89	Transects	2/ 3	Yes
CAR	Dzangha NP	499	2003	5	7.20	4.28	Transects	2/ 3	Yes
CAR	Ngotto	87	2002	0	158.40	0.80	Transects	7	
CAR	Ndoki NP	746	2003	56	26.00	2.92	Transects	2/ 3	Yes
Congo	Bailly	3,141	2010	50	56.00	0.85	Transects	1	Yes
Congo	Batanga	909	2007	15	42.00	0.13	Transects	1	Yes
Congo	Conkouati	3,452	2010	74	114.00	1.20	Transects	1	Yes
Congo	Dimonika	1,360	2008	0	65.00	1.41	Recces	5	
Congo	Foralac near Dimonika	1,988	2008	0	60.00	1.80	Recces	5	
Congo	Impfondo swamps	1,318	2008	7	44.00	0.11	Transects	1	
Congo	Kabo	2,870	2010	78	44.51	0.62	Transects	1	Yes
Congo	Lac tele marecage	2,107	2006	0	34.00	0.47	Transects	1	Yes
Congo	Lac Tele mixte	1,465	2006	0	42.00	0.98	Transects	1	Yes
Congo	Lac Tele TF	355	2006	0	30.00	0.57	Transects	1	Yes
Congo	Lefini	5,610	2006	221	713.00	0.53	Recces	1	Yes
Congo	Loundougou	4,320	2010	34	94.74	1.23	Transects	1	Yes
Congo	Mayoko	2,177	2005	48	159.00	3.69	Recces	1	
Congo	Mokabi	2,669	2006	3	29.00	4.33	Transects	1	
Congo	Mont Fouari	1,225	2007	102	167.00	0.21	Recces	1	
Congo	MPD	1,405	2009	99	202.00	1.13	Recces	1	Yes
Congo	Ngombe	11,016	2008	148	76.00	0.87	Transects	1	Yes
Congo	NNNP	4,190	2010	60	158.78	0.19	Transects	1	Yes
Congo	Ntokou	2,964	2008	49	35.88	0.20	Transects	1	Yes
Congo	Odzala Nord 2005	5,107	2005	102	30.00	0.43	Transects	1/ 8	Yes
Congo	Odzala Sud 2008	7,722	2008	318	62.34	0.24	Transects	1/ 8	Yes
Congo	Ogooue Leketi forest sector	4,302	2009	356	892.60	1.00	Recces	1	Yes
Congo	Pikounda	3,856	2008	79	35.85	0.17	Transects	1	Yes
Congo	Pokola	4,510	2010	240	94.91	2.39	Transects	1	Yes
Congo	Tanga	2,847	2009	19	153.20	1.64	Transects	1	
DRC	Bakwanza	1,449	2010	1	180.00	3.50	Recces	1	
DRC	Bili area	345	2005	85	160.00	0.18	Recces	9	
DRC	Hewa Bora	2,361	2007	4	71.00	0.90	Recces	1	

Country	Survey Site	Area (km^2)	Survey Year	Elephant Dung Piles (within 1m)	Effort (km)	Hunter Sign (/km)	Method	Source	Guards present
DRC	Itombwe	6,070	2007	8	1,038.00	0.41	Recces	1	Yes
DRC	Kahuzi Biega	6,600	2010	3	74.00	1.24	Transects	1	Yes
DRC	Mai Tatu	11,105	2009	8	619.00	2.09	Recces	1	
DRC	Maiko-Lubutu	1,000	2010	5	236.00	1.17	Recces	10	Yes
DRC	Maringa Lopori (RFLY)	3,465	2010	5	481.00	1.29	Recces	11	Yes
DRC	RFO Ituri	12,787	2011	76	143.00	1.36	Transects	1	Yes
DRC	Salonga	28,011	2004	21	125.60	0.70	Transects	1/ 3	Yes
DRC	Salonga Lokofa bloc	1,570	2010	125	205.00	2.56	Recces	1	Yes
DRC	Salonga NE end	1,590	2007	6	194.00	2.77	Recces	1	Yes
DRC	Salonga North corridor	400	2008	0	212.00	3.65	Recces	1	Yes
DRC	Salonga SE	1,739	2007	33	293.00	1.78	Recces	1	Yes
DRC	Salonga south corridor	391	2008	45	265.00	2.31	Recces	1	Yes
DRC	Sankuru west	6,900	2009	0	401.00	3.14	Recces	1	
DRC	Semliki Forest	220	2008	0	216.00	2.25	Recces	16	Yes
DRC	South Tumba	48	2011	0	86.20	3.65	Transects	12	
DRC	Tayna-Bunyuki & Mutenda	897	2006	30	61.00	0.84	Recces	13	Yes
DRC	TL2 north	5,447	2008	61	159.80	1.76	Transects	14	
DRC	TL2 south	12,437	2008	0	179.10	1.57	Transects	14	
DRC	Usala	1,152	2007	1	204.00	0.49	Recces	13	Yes
Gabon	Bateke	2,052	2006	427	586.00	2.89	Recces	1	Yes
Gabon	Birougou NP and buffer	1,985	2007	69	79.00	0.48	Transects	1	Yes
Gabon	Evaro	2,000	2005	223	170.00	0.89	Recces	1	
Gabon	Ivindo buffer zone (south)	863	2010	96	48.80	0.60	Transects	1	Yes
Gabon	Ivindo NP	2,967	2009	161	64.37	0.33	Transects	1	Yes
Gabon	Loango NP	1,510	2007	629	201.00	0.96	Transects	1/ 2	Yes
Gabon	Lope NP north	4,942	2009	227	55.00	0.37	Transects	1	Yes
Gabon	Lope NP south	4,942	2009	111	59.44	0.00	Transects	1	Yes
Gabon	Mayombe	1,200	2011	78	62.20	0.15	Transects	1	
Gabon	Mayumba NP	965	2010	106	50.45	1.05	Recces	1	Yes
Gabon	Minkebe high	2,301	2003	206	25.00	0.60	Transects	2/ 3	Yes
Gabon	Minkebe low	2,514	2003	184	16.00	0.00	Transects	2/ 3	Yes
Gabon	Minkebe medium	4,505	2003	155	19.00	0.21	Transects	2/ 3	Yes
Gabon	Monts de Cristal NP & buffer	5,300	2006	891	421.00	0.73	Recces	1	Yes
Gabon	Mwagne NP	1,167	2004	245	46.00	0.28	Recces	2/ 1	Yes
Gabon	Mwagne outside of park	454	2004	35	34.00	8.82	Recces	2/ 1	Yes
Gabon	Pongara NP	960	2006	70	36.50	2.40	Transects	1/ 15	Yes
Gabon	Waka	1,069	2006	1,239	344.00	0.16	Recces	1	Yes
Gabon	Waka-Lope corridor	2,991	2008	76	59.86	0.15	Transects	1	Yes

Sources: 1: Wildlife Conservation Society; 2: World Wide Fund for Nature; 3: MIKE-CITES; 4: Zoological Society Of San Diego; 5: JGI; 6: Projet CAF/95/G31-Foret de Bangassou; 7: Chimpanzee and Human Communication Institute, Central Washington University; 8: ECOFAC (EU); 9: Bili Project; 10: Fauna and Flora International; 11: African Wildlife Foundation; 12: University of Liege; 13: Diane Fossey Gorilla Fund International; 14: Lukuru Wildlife Research Foundation; 15: Especes Phares (EU) 16: Nixon S, Lusenge T (2008) Conservation status of okapi in Virunga National Park, Democratic Republic of Congo. London, UK: The Zoological Society of London. pp 54. Available: http://static.zsl.org/files/okapireport-compressed-656.pdf. Accessed 2013 Feb 20.
