# Supplementary material for: Devastating Decline of Forest Elephants in Central Africa
Source: PLoS One. 2013 Mar 4;8(3):e59469. doi: 10.1371/journal.pone.0059469 (PMC3587600; doi:10.1371/journal.pone.0059469)
Supplement: Table S2 — Analysis results for top-ranking predictive models (excluding hunter sign as an explanatory variable), which included (a) the Human Influence Index (HII), or (b) human population density and proximity to road (SPD). Details of the variables included in each model are given and percent deviance explained and UBRE score value. Estimated average elephant dung density (/km2) from model predictions across the Central African forests and bootstrapped 95% confidence intervals are shown. If the model included the survey year variable then prediction is for the endpoints of the time series (2002 and 2011); otherwise the prediction can be interpreted as an average over the 2002–2011 time period. Also shown for the models that permit temporal prediction is the overall percent decline and overall percent range loss for the period 2002–2011 (elephants are assumed to be absent when dung density falls below 100 elephant dung piles/km2; see Table S3 and Figure 2 for details, including a breakdown by country). (PDF) [file pone.0059469.s006.pdf]

Table S2. Analysis results for top-ranking predictive models (excluding hunter sign as an explanatory variable), which included (a) the Human Influence Index (HII), or (b) human population density and proximity to road (SPD). Details of the variables included in each model are given along with the percent deviance explained and UBRE score value. Estimated average elephant dung density (/km2) from model predictions across the Central African forests and bootstrapped 95% confidence intervals are shown. If the model included the survey year variable then prediction is for the endpoints of the time series (2002 and 2011); otherwise the prediction can be interpreted as an average over the 2002-2011 time period. Also shown for the models that permit temporal prediction is the overall percent decline and overall percent range loss for the period 2002-2011 (elephants are assumed to be absent when dung density falls below 100 elephant dung piles/km2; see Table S3 and Fig. 2 for details, including a breakdown by country).

Model Group	Model No.	Model Variables	Deviance explained	UBRE score	Prediction Period	Average Dung Density	95% CI	% Decline	% Range Loss
**a**	HII 1	HumanInfluence.Corruption.Guards.Lon	41.4%	1.8450	2002-2011	175.56	(118.63 - 280.85)	-	
	HII 2	Year.HumanInfluence.Corruption.Guards.Lat.Lon	46.1%	1.8479	2002	271.80	(115.29 - 751.07)	55.30	17.68
					2011	121.49	(64.08 - 345.16)		
	HII 3	HumanInfluence.Corruption.Guards	40.8%	1.8614	2002-2011	178.18	(116.01 - 241.30)	-	
	HII 4	Year.HumanInfluence.Corruption.Guards.Lon	43.1%	1.9099	2002	327.06	(146.50 - 776.32)	69.12	29.84
					2011	101.00	(53.60 - 253.45)		
	HII 5	Year.HumanInfluence.Corruption.Guards	41.1%	1.9279	2002	347.78	(116.25 - 648.40)	69.04	31.33
					2011	107.67	(53.84 - 202.00)		
**b**	SPD 1	Year.SitePopDensity.Guards.Lat.Lon	51.6%	1.5764	2002	285.85	(111.24 - 913.29)	51.33	22.37
					2011	139.11	(60.50 - 454.26)		
	SPD 2	Year.SitePopDensity.Corruption.Guards	43.3%	1.5888	2002	299.90	(108.62 - 664.36)	62.11	41.57
					2011	113.63	(54.55 - 290.61)		
	SPD 3	Dist2Road.SitePopDensity.Corruption.Guards	43.6%	1.6506	2002-2011	174.61	(118.63 - 292.76)	-	
	SPD 4	Year.Dist2Road.SitePopDensity.Corruption.Guards	44.9%	1.6616	2002	298.95	(52.64 - 661.03)	63.59	37.26
					2011	108.86	(50.74 - 282.28)		
	SPD 5	Dist2Road.SitePopDensity.Guards.Lat.Lon	52.3%	1.7652	2002-2011	205.34	(115.29 - 442.11)	-	
	SPD 6	Year.Dist2Road.SitePopDensity.Corruption.Guards. Lat.Lon	45.6%	1.8554	2002	283.23	(76.23 - 797.04)	60.89	27.69
					2011	110.77	(55.03 - 506.43)		
							**Average**	**61.63**	**29.68**
