# Supplementary material for: Devastating Decline of Forest Elephants in Central Africa
Source: PLoS One. 2013 Mar 4;8(3):e59469. doi: 10.1371/journal.pone.0059469 (PMC3587600; doi:10.1371/journal.pone.0059469)
Supplement: Table S3 — Estimates of percentage extremely low density elephant range across the Central African forests and by country (relative to each country’s forested area) for 2002 and 2011 for the top-ranking predictive models, which included the survey year variable. Elephants are assumed to be almost absent when dung density falls below a threshold value of 100 elephant dung piles/km2. Also shown are estimates of the percentage of potential habitat at high elephant density (defined as >1,000 elephant dung piles/km2). The average across all models for 2002 and for 2011 is shown, as well as the range Table S6 for a breakdown of forest cover by country. (PDF) [file pone.0059469.s007.pdf]

Table S3. Estimates of percentage extremely low density elephant range across the Central African forests and by country (relative to each country's forested area) for 2002 and 2011 for the top-ranking predictive models, which included the survey year variable. Elephants are assumed to be almost absent when dung density falls below a threshold value of 100 elephant dung piles/km^2. Also shown are estimates of the percentage of potential habitat at high elephant density (defined as > 1,000 elephant dung piles/km^2). The average across all models for 2002 and for 2011 is shown, as well as the range Table S6 for a breakdown of forest cover by country.

Threshold	Model No.	Prediction Period	Estimate of extremely low density elephant range (%)					
			**Overall**	**Cameroon**	**CAR**	**DRC**	**Congo**	**Gabon**
< 100	HII 2	2002	54.77	29.11	35.85	78.09	13.27	4.09
		2011	72.45	53.72	61.45	95.57	38.33	8.53
	HII 4	2002	48.05	28.77	43.85	68.70	7.64	1.52
		2011	77.89	79.29	84.14	95.99	53.45	10.94
	HII 5	2002	46.99	30.48	28.44	67.39	9.24	0.57
		2011	78.33	80.25	74.88	95.68	60.35	10.47
	SPD 1	2002	46.43	16.11	53.15	68.14	5.39	0.19
		2011	68.80	31.70	88.82	90.73	46.06	4.18
	SPD 2	2002	37.11	19.25	3.33	55.83	2.74	0.06
		2011	78.68	84.58	89.71	94.44	72.78	0.07
	SPD 4	2002	39.83	19.41	3.26	60.08	3.11	0.06
		2011	77.09	81.32	86.55	94.51	63.03	0.17
	SPD 6	2002	48.41	17.17	3.16	74.12	3.05	0.06
		2011	76.11	74.44	81.59	94.85	59.64	0.10
	Average	**2002**	45.94	22.90	24.43	67.48	6.35	0.94
		**2011**	75.62	69.33	81.02	94.54	56.24	4.92
		2011-2002	***-29.68***	***-46.43***	***-56.58***	***-27.06***	***-49.89***	***-3.99***
		**% habitat at high elephant density**						
> 1,000	HII 2	2002	6.34	8.23	2.48	0.00	10.41	33.19
		2011	2.11	0.00	0.00	0.00	0.00	16.66
	HII 4	2002	9.07	7.44	2.38	0.00	21.21	44.25
		2011	1.38	0.00	0.00	0.00	0.00	10.88
	HII 5	2002	7.66	4.22	2.49	0.00	11.99	44.67
		2011	1.83	0.00	0.00	0.00	0.00	14.45
	SPD 1	2002	5.87	10.15	0.00	0.00	8.66	30.54
		2011	1.96	0.05	0.00	0.00	0.37	15.05
	SPD 2	2002	3.38	0.00	9.32	0.00	0.00	24.43
		2011	2.89	0.00	0.00	0.00	0.00	22.80
	SPD 4	2002	5.19	2.99	2.06	0.00	11.38	26.81
		2011	1.25	0.00	0.00	0.00	0.00	9.85
	SPD 6	2002	5.20	4.10	2.01	0.00	11.10	26.39
		2011	1.17	0.00	0.00	0.00	0.00	9.23
	Average	**2002**	6.10	5.31	2.96	0.00	10.68	32.90
		**2011**	1.80	0.01	0.00	0.00	0.05	14.13
		2011-2002	***-4.31***	***-5.30***	***-2.96***	***0.00***	***-10.63***	***-18.77***
