# Supplementary material for: Devastating Decline of Forest Elephants in Central Africa
Source: PLoS One. 2013 Mar 4;8(3):e59469. doi: 10.1371/journal.pone.0059469 (PMC3587600; doi:10.1371/journal.pone.0059469)
Supplement: Table S4 — Description of spatial variables, data source, method of calculation, likely influence on elephant density, UBRE score and deviance explained for the single variable models. (PDF) [file pone.0059469.s008.pdf]

Table S4. Description of spatial variables, data source, method of calculation, likely influence on elephant density, UBRE score and deviance explained for the single variable models.

**Variable Type**	**Variable Name**	**Description**	**Data Source**	**Method of calculating Variable value**	**Potential influence on elephant density**	**UBRE score**	**Deviance explained**
Survey Site Specific	Hunter Sign	Encounter rate of all sign associated with hunting, such as snares, shotgun cartridges, camps, machete cuts, hunter paths.	Survey datasets and reports	Number of signs per kilometre walked.	Elephant density negatively associated with increasing hunter sign encounter rate.	1.9283	14.9%
	Year	Calendar year.	Survey datasets and reports	Calendar year survey was conducted.	Elephant density negatively associated with surveys completed in recent years.	1.8268	7.7%
	Dist2Road	Proximity to roads.	World Conservation Monitoring Centre dataset and local adjustments for errors	Average value across the survey site in projected Universal Transverse Mercator (UTM) coordinates.	Elephant density positively associated with increasing distance away from roads.	1.9713	7.9%
	SitePop Density	Number of people per square kilometre.	Gridded Population of the World http://sedac.ciesin.columbia.edu/gpw/	Average value across the survey site using the 2005 data layer for surveys completed 2002-2007 and the 2010 data layer for those completed 2008-2011.	Elephant density negatively associated with sites with a higher overall human population density.	2.1513	25.2%
	Human Influence Index	Aggregate score for suite of variables (see reference).	Last of the Wild Data Version 2 http://sedac.ciesin.columbia.edu/wildareas/downloads.jsp#infl	Average value across the survey site.	Elephant density negatively associated with increasing Human Influence Index.	2.4976	14.5%
	Official Protection	Score reflecting degree of protection.	Central African Regional Program for the Environment (CARPE).	Ranges from 1 (National Park) to 5 (no official protection).	Elephant density positively associated with sites where official protection is stronger.	3.3039	9.4%
	Guards	Whether or not a site has guards.	Knowledge of the sites.	0 or 1.	Elephant density positively associated with sites where wildlife guards are employed.	2.1463	14.2%
Country Specific	Country	Name of country.	NA	Unique value assigned per country.	Elephant density positively associated with more developed, less corrupt countries where conservation is higher on the agenda.	1.7829	32.0%
	Corruption	Degree of corruption.	Transparency International's Corruption Perceptions Index 2006 http://www.transparency.org/policy_research/surveys_indices/cpi	Ranges from 0-10. Smaller values indicate higher levels of corruption.	Elephant density negatively associated with a l ower Corruption Perception Index (corresponding to higher levels of corruption).	1.7742	31.4%
Regional Proxies	Lat	Latitude coordinate.	NA	Approximate centroid of each survey site.	NA	2.1553	9.5%
	Lon	Longitude coordinate.	NA	Approximate centroid of each survey site.	NA	1.5754	31.0%
