# Supplementary material for: Devastating Decline of Forest Elephants in Central Africa
Source: PLoS One. 2013 Mar 4;8(3):e59469. doi: 10.1371/journal.pone.0059469 (PMC3587600; doi:10.1371/journal.pone.0059469)
Supplement: Table S5 — Analysis results for top-ranking models which included the hunter sign variable. Hunter sign was not included in the predictive model across the Central African forests, as it was unavailable at that scale. (PDF) [file pone.0059469.s009.pdf]

Table S5. Analysis results for top-ranking models which included the hunter sign variable. Hunter sign was not included in the predictive model across the Central African forests, as it was unavailable at that scale.

**Model Variables**	**UBRE score**	**Deviance explained**
HunterSign.Year.Dist2Road.SitePopDensity.Corruption.Guards	1.3279	48.50%
HunterSign.HumanInfluence.Guards.Lat.Lon	1.4838	54.00%
HunterSign.Year.Dist2Road.SitePopDensity.Corruption.Guards.Lat.Lon	1.6204	50.00%
HunterSign.YearByCountry.Dist2Road.SitePopDensity.Corruption.Guards	1.6545	53.90%
HunterSign.YearByCountry.SitePopDensity.Corruption.Guards	1.6736	51.90%
HunterSign.Year.HumanInfluence.Corruption.Guards	1.7051	46.50%
HunterSign.Year.HumanInfluence.Corruption.Guards.Lat.Lon	1.7656	50.20%
HunterSign.YearByCountry.HumanInfluence.Corruption.Guards	2.0261	48.70%
