# Supplementary material for: Devastating Decline of Forest Elephants in Central Africa
Source: PLoS One. 2013 Mar 4;8(3):e59469. doi: 10.1371/journal.pone.0059469 (PMC3587600; doi:10.1371/journal.pone.0059469)
Supplement: Table S6 — Estimated forest cover by country as defined by Iremonger et al. (1997) [96] . (PDF) [file pone.0059469.s010.pdf]

Table S6. Estimated forest cover by country as defined by Iremonger et al. (1997) [96].

| **Country** | **Forest cover ($km^2$)** | **Percent of total forest cover** |
|---|---|---|
| Cameroon | 191,536 | 8.67% |
| Central African Republic | 67,394 | 3.05% |
| Democratic Republic of Congo | 1,383,517 | 62.64% |
| Republic of Congo | 286,454 | 12.97% |
| Gabon | 279,813 | 12.67% |
